# Acceptability of COVID-19 Vaccine Among Hospital Employees in the Department of Paediatrics, Gynaecology and Obstetrics in the University Hospitals of Geneva, Switzerland

**DOI:** 10.3389/fpubh.2021.781562

**Published:** 2022-01-27

**Authors:** Anna Peirolo, Klara M. Posfay-Barbe, Dominic Rohner, Noémie Wagner, Geraldine Blanchard-Rohner

**Affiliations:** ^1^Children's Hospital of Brescia, Brescia, Italy; ^2^Paediatric Immunology and Vaccinology Unit, Division of General Paediatrics, Department of Paediatrics, Gynaecology and Obstetrics, Geneva University Hospitals and University of Geneva, Geneva, Switzerland; ^3^Paediatric Infectious Diseases Unit, Division of General Paediatrics, Department of Paediatrics, Gynaecology and Obstetrics, Geneva University Hospitals and University of Geneva, Geneva, Switzerland; ^4^Department of Economics, Faculty of Business and Economics Hautes études commerciales (HEC) Lausanne), University of Lausanne and Centre for Economic Policy Research (CEPR), Lausanne, Switzerland

**Keywords:** COVID-19, vaccine, Paediatrics, Gynaecology, Obstetrics, health care workers

## Abstract

**Background and Aims:**

COVID-19 vaccination has been in the spotlight for almost a year now, both within the scientific community and in the general population. The issue of healthcare workers' (HCWs) hesitancy is particularly salient, given that they are at the forefront of the fight against COVID-19. Not only could unvaccinated HCW spread the disease, but HCWs are also critical messengers in building confidence towards COVID-19 vaccines. The goal of this study was to examine the perception of COVID-19 risk and of its vaccine acceptance among employees (i.e., HCW plus administrative staff) in the Department of Paediatrics, Gynaecology and Obstetrics at the University Hospitals of Geneva, for the purpose of drawing lessons on the determinants of vaccination morale.

**Methods:**

We conducted an anonymous online survey comparing vaccination attitudes among vaccinated and unvaccinated workers in June 2021. It included questions on perception of COVID-19 risks and COVID-19 vaccines. Vaccination was not mandatory in our institution but was strongly recommended.

**Results:**

In June 2021, 66% of the 1,800 employees of our department had received two doses of COVID-19 vaccine by the time of the survey. Among the employees, 776 participated (43%) to the survey, and among them 684 (88%) had chosen to be vaccinated. Participants working for longer in a hospital, with a chronic disease and a household contact with chronic disease were more likely to be vaccinated. Doctors were twice as likely to be vaccinated than nurses. Among unvaccinated hospital employees, 48 (52%) responded that they would not change their mind. Further, 35 (38%) were not feeling in danger of contracting severe COVID-19, and 32 (35%) had fears about possible side effects of COVID-19 vaccines that they wanted to discuss with a specialist.

**Conclusion:**

Our study indicates that, while two-third of the employees had been vaccinated, quite many were still hesitant. The unvaccinated explained their choice by not feeling at risk of complicated COVID-19, and because of fear of possible side effects associated with the vaccine. Investments in COVID-19 vaccine education is a critical component for increasing vaccine acceptance among the unvaccinated.

## Introduction

In January 2020 the World Health Organisation (WHO) announced the outbreak in China of a new coronavirus, which later on became known as severe acute respiratory syndrome coronavirus 2 (SARS-CoV-2) leading to the COVID-19 disease. Since then COVID-19 has spread around the globe, causing to date over 276 million confirmed cases and over 5.3 million deaths (WHO numbers, 23 December 2021) (1, 2). The first COVID-19 vaccines were globally commercialised at the beginning of December 2020, with the messenger RNA-based (mRNA) vaccine of Pfizer-BioNTech (COMIRNATY) being the first authorised in the United Kingdom on December 3, 2020, and in Switzerland on December 19, 2020 (3). The mRNA-1273 vaccine (Moderna) was approved in Switzerland on the January 12, 2021. By the time the COVID-19 vaccination campaign had started in Switzerland (December 23, 2020), there had been 6,406 deaths reported in Switzerland since the beginning of the pandemic (3). At that time, 21% of the Geneva canton's population had been infected with SARS-CoV-2, suggesting a relatively slow rise in population immunity (3). Adults ≥65 years old, younger persons with comorbidities, or people living in close contact with these categories, and healthcare workers (HCWs) were considered the priority groups for COVID-19 vaccination (3). Subsequently the vaccination was extended to the rest of the population aged 16–64 years. By June 22, 2021, Pfizer vaccine was also authorised in children aged 12–15 (3).

Reaching sufficient coverage is dependent on both the vaccines' effectiveness and people's willingness to be vaccinated (4, 5). However, along with increased vaccine use and popularity, there have also been public concerns about their safety and efficacy. This loss of confidence, known as “vaccine hesitancy,” have now been explored across the world and it is fairly well-established that it involves general population as well as healthcare workers (HCWs) (1, 2, 6, 7). From the beginning of the COVID-19 pandemic, HCWs have demonstrated professional dedication despite the fear of becoming infected and infecting patients or family members (8). They also represent a trusted source of information on vaccination for the general population, and can shield against misleading and confusing information (9). Given the need for identifying factors associated with vaccine acceptance and hesitancy for implementing immunisation policy (9, 10), we have conducted a novel Swiss survey on the perception of COVID-19 risk and vaccine acceptance among hospital employees more than 1 year after the beginning of the COVID-19 pandemic.

## Materials and Methods

### Study Design and Population

This single centre, cross-sectional study enrolled all employees of the Department of Paediatrics, Gynaecology and Obstetrics (DPGO) part of the University Hospitals of Geneva (HUG) regardless of the professional category or setting in which they were working.

An anonymous open online questionnaire was sent to all professional email addresses between the 1st and the 30th of June, 2021. No incentives were offered, and the email recipients were informed about the expected length of the survey. At the time of the survey, two mRNA-based vaccines were available in the canton of Geneva—the Comirnaty^®^ (BNT162b2) vaccine of Pfizer/BioNTech and the COVID-19 vaccine (mRNA-1273) of Moderna vaccines (3). No exclusion criterion was applied; willingness to complete the questionnaire was the only requirement to participate to the survey. The Regional Ethics Committee [“Commission Cantonale d'Ethique de la Recherche sur l'être humain” (CCER)] has approved the study (CCER 2021-00838). The data was saved in a password-protected file stocked on the server of the hospital.

### Questionnaire

The questionnaire was developed based on a previous study on influenza vaccine acceptance during pregnancy (11) and according to a literature review. It was composed by 42 questions, with a “yes and no” answer form or 5-point rating scale, grouped into four sections (see Appendix in the Supplementary Material). Section Introduction intended to evaluate the perception regarding SARS-CoV-2 infection in general and in specific risk groups. Section Materials and Methods contained questions about COVID-19 vaccine perception. Section Results queried about the vaccination status and was composed by 9 and 11 questions for the vaccinated and non-vaccinated, respectively, exploring the reasons for personal decision. Section Discussion consisted of demographic questions. Participants were informed that answering the questions was voluntary and anonymous, and that the results would be scientifically evaluated and published. The questionnaire was developed electronically on SurveyMonkey (Survey-Monkey Inc., San Mateo, CA, USA) and distributed over a 4-week period via a mailing list. Over the course of the survey period, one reminder was sent out through email to all employees.

### Statistical Analysis

Categorical variables were described in numbers and percentages, while numerical variables in mean and standard deviation. The answers between the 2 groups (vaccinated and unvaccinated hospital employees) were compared using the Chi-square test. We also tested the factors increasing the likelihood of being immunised using an univariate logistic regression analysis, and we reported odds ratio (OR). An OR >1 corresponded to an increase in the probability to be vaccinated, while an OR <1 corresponded to a decreased likelihood to be vaccinated. A two-sided *P* < 0.05 was considered statistically significant.

## Results

**Figure 1:**
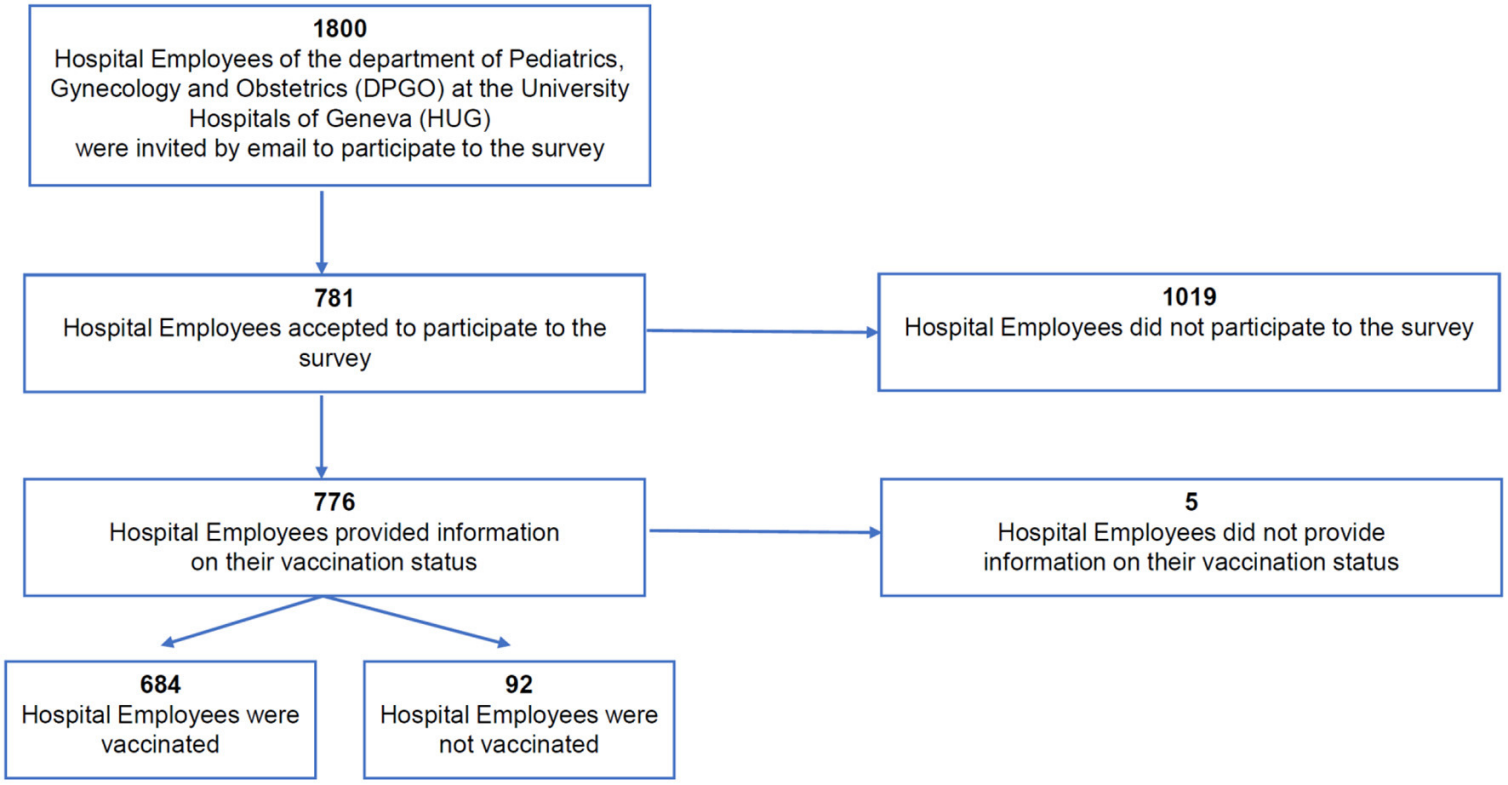
Flowchart of the study population.

A total of 781 out of the around 1,800 members of the HUG accessed the survey and 776 (43%) gave information on their vaccination status and were considered for analysis. All employees were divided into two groups: SARS-CoV-2 vaccinated (*n* = 684, 88%) and unvaccinated (*n* = 92, 12%) (Figure 1).

### Demographic Data

The demographic data are shown in Table 1. Most participants were female (*n* = 651, 84%). Four hundred sixty-eight (60%) participants were working for >10 years in a hospital. Those working in a hospital for 1–5 years were twice less likely to be vaccinated than those working in a hospital for >10 years (OR = 0.5, *p* = 0.025). In terms of professional category, the largest group was represented by the nurses (43%), followed by doctors (27%), administrative staff (12%), respiratory/physical/speech therapists (7%), auxiliary nursing staff (4%), patient care technicians (1%), other category (3%, such as education teacher, biologist, research, and laboratory staff), and social workers (0.3%). A significant difference in vaccine status was noted between the professional categories, with doctors being twice more likely to be vaccinated in comparison to nurses (OR = 2.4, *p* = 0.009).

**Table 1:** Characteristics of the study population.

**Characteristic**	**Total^a^**	**Vaccinated**	**Non-vaccinated**	***p*-values^b^**	**Odds ratio**	**(*p*-value)**
	**(*N* = 776, 100%)**	**(*N* = 684, 88%)**	**(*N* = 92, 12%)**	**(Chi^**2**^-test)**	**(Regression analysis)**	
Sex: *N* (%)				**<0.001**		
Female	651 (84%)	574 (88%)	77 (12%)		Reference category	
Male	102 (13%)	96 (94%)	6 (6%)		2.1	(*p* = 0.081)
Missing information	23 (3%)	14 (61%)	9 (39%)		NA	
No of years working at the HUG: *N* (%)				**0.001**		
>10 years	468 (60%)	423 (90%)	45 (10%)		Reference category	
6–10 years	135 (17%)	121 (90%)	14 (10%)		0.9	(*p* = 0.795)
1–5 years	132 (17%)	110 (83%)	22 (17%)		**0.5**	**(*****p*** **=** **0.025)**
<1 year	19 (3%)	16 (84%)	3 (16%)		0.6	(*p* = 0.382)
Missing information	22 (3%)	14 (2%)	8 (9%)		0.2	(*p* < 0.001)
Professional category: *N* (%)				**0.001**		
Nurse	332 (43%)	289 (87%)	43 (13%)		Reference category	
Doctor	208 (27%)	196 (94%)	12 (6%)		**2.4**	**(*****p*** **=** **0.009)**
Auxiliary nursing staff	34 (4%)	27 (79%)	7 (21%)		0.6	(*p* = 0.222)
Patient care technician	4 (1%)	4 (100%)	0 (0%)		NA	
Administrator staff	95 (12%)	82 (86%)	13 (14%)		0.9	(*p* = 0.852)
Respiratory, physical, or speech therapist	55 (7%)	50 (91%)	5 (9%)		1.5	(*p* = 0.424)
Social workers	2 (0%)	1 (50%)	1 (50%)		0.2	(*p* = 0.181)
Other category^c^	24 (3%)	21 (88%)	3 (13%)		1.0	(*p* = 0.949)
Missing information	22 (3%)	14 (64%)	8 (36%)		NA	
Working with risk groups: *N* (%)				0.439		
None	141 (18%)	121 (86%)	20 (14%)		Reference category	
New born	21 (3%)	17 (2%)	4 (4%)		0.9	(*p* = 0.844)
Toddler	18 (2%)	17 (2%)	1 (1%)		3.6	(*p* = 0.225)
Immunocompromised patients (IP)	36 (5%)	36 (5%)	0 (0%)		NA	
Pregnant	70 (9%)	62 (9%)	8 (9%)		1.6	(*p* = 0.254)
Toddler and IP	19 (2%)	18 (3%)	1 (1%)		3.8	(*p* = 0.205)
New born and IP	4 (0.5%)	4 (1%)	0 (0%)		NA	
New born and toddler	35 (5%)	31 (5%)	4 (4%)		1.6	(*p* = 0.393)
Pregnant and new born	92 (12%)	81 (12%)	11 (12%)		1.5	(*p* = 0.251)
Pregnant and toddler	9 (1%)	9 (1%)	0 (0%)		NA	
Pregnant and IP	10 (1%)	9 (1%)	1 (1%)		1.9	(*p* = 0.554)
All risk groups patients	106 (14%)	93 (14%)	13 (14%)		1.5	(*p* = 0.256)
New born, pregnant, and IP	19 (2%)	17 (2%)	2 (2%)		1.8	(*p* = 0.456)
New born, toddler, and IP	37 (5%)	33 (5%)	4 (4%)		1.7	(*p* = 0.333)
Pregnant, new born, and toddler	117 (15%)	105 (15%)	12 (13%)		1.8	(*p* = 0.095)
Pregnant, toddler, and IP	4 (0.5%)	4 (1%)	0 (0%)		NA	
Missing information	38 (5%)	27 (4%)	11 (12%)		NA	
Chronic disease: *N* (%)				**0.038**		
Nobody	518 (67%)	459 (89%)	59 (11%)		Reference category	
Yes, me	65 (8%)	58 (89%)	7 (11%)		1.1	(*p* = 0.882)
Yes, my household contacts	156 (20%)	139 (89%)	17 (11%)		1.1	(*p* = 0.865)
Me and my household contacts	12 (2%)	11 (92%)	1 (8%)		**1.7**	**(*****p*** **=** **0.042)**
Missing information	25 (3%)	17 (68%)	8 (32%)		0.3	(*p* = 0.004)
Living with children: *N* (%)				0.059		
No kids	277 (36%)	248 (90%)	29 (10%)		Reference category	
<12 years	200 (26%)	175 (88%)	25 (13%)		0.8	(*p* = 0.490)
12–18 years	85 (11%)	72 (85%)	13 (15%)		0.6	(*p* = 0.227)
>18 years	93 (12%)	85 (91%)	8 (9%)		1.2	(*p* = 0.604)
12–18 and >18 years	37 (5%)	34 (92%)	3 (8%)		1.3	(*p* = 0.657)
<12 and >18 years	3 (0%)	3 (100%)	0 (0%)		NA	
<12 and 12–18 years	48 (6%)	44 (92%)	4 (8%)		1.3	(*p* = 0.652)
All categories	8 (1%)	6 (75%)	2 (25%)		0.4	(*p* = 0.212)
Missing information	25 (3%)	17 (68%)	8 (32%)		0.2	(*p* < 0.001)

a *Five persons (out of the total of 781 who responded to the questionnaire) did not indicate if they had been vaccinated or not and were excluded from analysis*.

b *p < 0.05 indicates a significant difference (highlighted in bold)*.

c *Other category: education teacher, biologist, research, and laboratory staff*.

Among the study participants, 597 (77%) worked in contact with patients who are vulnerable and/or at high risk of severe COVID-19 (new-borns, toddlers, immunocompromised patients, and/or pregnant women). There was no significant difference (*p* = 0.439) in the choice of vaccination between the hospital employees working with different risk groups' patients. Thirty percent of respondents (*n* = 233) reported having a chronic disease and/or to have vulnerable persons as household contacts. The persons with chronic disease and with household contacts with chronic disease were almost twice more likely to be vaccinated compared to those in good health and not living with at risk household contacts (OR = 1.7, *p* = 0.042). Among study participants, 474 (61%) had at least one child. Having children did not influence the likelihood to be vaccinated.

### Perception on COVID-19 Infection

Respondents who thought that the COVID-19 infection could be very severe in adults were 12.5 times more likely to be vaccinated, in comparison to those who thought that COVID-19 was not at all severe (OR = 12.5, *p* = 0.01). Similarly, those who thought that COVID-19 could be very severe in infants and children were 9.1 times more likely to be vaccinated compared to those who thought that COVID-19 was not at all severe in children (OR = 9.1, *p* = 0.004). Again, those who thought that COVID-19 could be severe in pregnant women were 8 times more likely to be vaccinated than those who believed that COVID-19 was not at all severe in pregnant women (OR = 8.2, *p* < 0.001). Finally, the respondents who thought that COVID-19 could be very severe in at risk patients were 7 times more likely to be vaccinated than those who thought that COVID-19 was not at all severe in this group of patients (OR = 7.0, *p* = 0.036).

Five hundred seventy participants (56%) perceived their workplace as representing a risk for being infected, while 123 (16%) believed the opposite, and 199 (26%) had no opinion. The latter ones were less likely to be vaccinated than the first ones. Four hundred fifty-nine participants (60%) considered themselves as potential spreaders of the infection for patients, while 120 persons (15%) disagreed. The second group was less likely to be vaccinated. Six hundred twelve participants (79%) considered themselves as possible spreaders for household contacts, compared to 57 (7%) who disagreed. The second group was less likely to be vaccinated (see Table 2 for all *p*-values).

**Table 2:** Perception on COVID-19 disease.

	**Total^a^**	**Vaccinated**	**Non-vaccinated**	***p*-values^b^**	**Odds ratio**	**(*p*-value)**
**Question**	**(*N* = 776, 100%)**	**(*N* = 684, 88%)**	**(*N* = 92, 12%)**	**(Chi^**2**^-test)**	**(Regression analysis)**
COVID-19 in adults: *N* (%)				**<0.001**		
Not at all severe	6 (1%)	4 (67%)	2 (33%)		Reference category
Not severe	11 (1%)	7 (64%)	4 (36%)		0.875	(*p* = 0.901)
Mild	200 (26%)	155 (78%)	45 (23%)		1.7	(*p* = 0.538)
Severe	426 (55%)	390 (92%)	36 (8%)		5.5	(*p* = 0.056)
Very severe	130 (17%)	125 (96%)	5 (4%)		**12.5**	**(*****p*** **=** **0.010)**
Missing information	3 (0%)	3 (100%)	0 (0%)		NA	
COVID-19 in children: *N* (%)				**<0.001**		
Not at all severe	85 (11%)	67 (79%)	18 (21%)		Reference category
Not severe	130 (17%)	106 (82%)	24 (18%)		1.2	(*p* = 0.624)
Mild	281 (36%)	245 (87%)	36 (13%)		1.8	(*p* = 0.059)
Severe	197 (25%)	185 (94%)	12 (6%)		**4.2**	**(*****p*** **<** **0.001)**
Very severe	70 (9%)	68 (97%)	2 (3%)		**9.1**	**(*****p*** **=** **0.004)**
Missing information	13 (2%)	13 (100%)	0 (0%)		NA	
COVID-19 in pregnant women: *N* (%)			**<0.001**		
Not at all severe	20 (3%)	12 (60%)	8 (40%)		Reference category
Not severe	43 (5%)	35 (81%)	8 (19%)		2.9	(*p* = 0.075)
Mild	245 (32%)	209 (85%)	36 (15%)		**3.9**	**(*****p*** **=** **0.006)**
Severe	344 (44%)	318 (92%)	26 (8%)		**8.2**	**(*****p*** **<** **0.001)**
Very severe	113 (15%)	99 (88%)	14 (12%)		**4.7**	**(*****p*** **=** **0.004)**
Missing information	11 (1%)	11 (100%)	0 (0%)		NA	
COVID-19 in patients with chronic disease or with risk factors^c^: *N* (%)				**<0.001**		
Not at all severe	5 (1%)	3 (60%)	2 (40%)		Reference category
Not severe	0	0	0		NA	
Severe	26 (3%)	15 (58%)	11 (42%)		0.9	(*p* = 0.924)
Mild	198 (25%)	166 (84%)	32 (16%)		3.5	(*p* = 0.184)
Very severe	540 (70%)	493 (91%)	47 (9%)		**7.0**	**(*****p*** **=** **0.036)**
Missing information	7 (1%)	7 (100%)	0 (0%)		NA	
Workplace as high risk of exposure: *N* (%)			**0.011**		
Absolutely agree	273 (35%)	250 (92%)	23 (8%)		Reference category
Agree	159 (20%)	144 (91%)	15 (9%)		0.9	(*p* = 0.721)
Indifferent	199 (26%)	163 (82%)	36 (18%)		**0.4**	**(*****p*** **=** **0.002)**
Disagree	91 (12%)	82 (90%)	9 (10%)		0.8	(*p* = 0.669)
Absolutely disagree	32 (4%)	25 (78%)	7 (22%)		**0.3**	**(*****p*** **=** **0.020)**
Missing information	22 (3%)	20 (91%)	2 (9%)		0.9	(*p* = 0.914)
If infected, perceive myself as possible spreader to patients I treat: *N* (%)				**<0.001**		
Absolutely agree	314 (41%)	289 (92%)	25 (8%)		Reference category
Agree	145 (19%)	131 (90%)	14 (10%)		0.8	(*p* = 0.546)
Indifferent	158 (20%)	129 (82%)	29 (18%)		**0.4**	**(*****p*** **=** **0.001)**
Disagree	80 (10%)	69 (86%)	11 (14%)		0.5	(*p* = 0.113)
Absolutely disagree	40 (5%)	28 (70%)	12 (30%)		**0.2**	**(*****p*** **<** **0.001)**
Missing information	39 (5%)	38 (97%)	1 (3%)		3.3	(*p* = 0.250)
If infected, perceive myself as possible spreader to my family: *N* (%)				**<0.001**		
Absolutely agree	480 (62%)	433 (90%)	47 (10%)		Reference category
Agree	132 (17%)	115 (87%)	17 (13%)		0.7	(*p* = 0.306)
Indifferent	56 (7%)	41 (73%)	15 (27%)		**0.3**	**(*****p*** **<** **0.001)**
Disagree	34 (4%)	25 (74%)	9 (27%)		**0.3**	**(*****p*** **=** **0.004)**
Absolutely disagree	23 (3%)	20 (87%)	3 (13%)		0.7	(*p* = 0.612)
Missing information	51 (7%)	50 (98%)	1 (2%)		5.4	(*p* = 0.098)

a *Five persons (out of the total of 781 who responded to the questionnaire) did not indicate if they had been vaccinated or not and were excluded from analysis*.

b *p < 0.05 indicates a significant difference (highlighted in bold)*.

c *Seniors (≥65 years), immunocompromised people, people suffering from chronic conditions*.

### Perception on COVID-19 Vaccine

Five hundred and seventieth persons (74%) participants believed that COVID-19 vaccine prevents from severe forms of the disease, while 63 (8%) disagreed with this statement, and 102 (13%) had no opinion. Those who did not absolutely agree with this statement were less likely to be vaccinated. Five hundred forty-four persons (70%) believed that COVID-19 vaccine would be a cornerstone to end the pandemic, while 70 (9%) disagreed. Those who did not absolutely agree with this statement were less likely to be vaccinated. One hundred eight (14%) believed that COVID-19 vaccines could lead to stop taking precautions after being vaccinated (i.e., stop wearing a mask, social distancing), while 446 (57%) disagreed with this information, with no statistical difference in vaccine acceptance between both groups. There was no difference in vaccination perception on how the COVID-19 vaccine could help to stop taking precautions. Four hundred six participants (59%) believed that the vaccine is safe, while 90 (12%) disagreed, and 251 (32%) had no opinion. The two latter groups were less likely to be vaccinated, compared to those who absolutely agreed that the vaccine was safe. Four hundred seventy-six participants (71%) did not feel protected by previous COVID-19 infection, while 87 (11%) disagreed, and 167 (22%) had no opinion. The two latter groups were less likely to be vaccinated compared to those who absolutely agreed that they were not feeling protected by previous COVID-19 infection. Two hundred ninety-nine (39%) participants believed that the vaccine does protect against the latest COVID-19 variants, while 338 (44%) did not know, and 128 (16%) disagreed. The two latter groups were less likely to be vaccinated compared to those who absolutely agreed that COVID-19 vaccines protect against SARS-CoV-2 variants (see Table 3).

**Table 3:** Perception on COVID-19 vaccine.

	**Total^a^**	**Vaccinated**	**Non-vaccinated**	***p*-values^b^**	**Odds ratio**	**(*p*-value)**
**Question**	**(*N* = 776, 100%)**	**(*N* = 684, 88%)**	**(*N* = 92, 12%)**	**(Chi^**2**^-test)**	**(Regression analysis)**
COVID-19 vaccine prevents severe forms of the disease: *N* (%)		**<** **0.001**		
Absolutely agree	434 (56%)	413 (95%)	21 (5%)		Reference category
Agree	136 (18%)	115 (85%)	21 (15%)		**0.3**	**(*****p*** **<** **0.001)**
Indifferent	102 (13%)	71 (70%)	31 (31%)		**0.1**	**(*****p*** **<** **0.001)**
Disagree	35 (4%)	10 (71%)	10 (29%)		**0.1**	**(*****p*** **<** **0.001)**
Absolutely disagree	28 (4%)	9 (68%)	9 (32%)		**0.1**	**(*****p*** **<** **0.001)**
Missing information	41 (5%)	41 (100%)	0 (0%)		NA	
COVID-19 vaccines could be a cornerstone to end the pandemic: *N* (%)				**<** **0.001**		
Absolutely agree	402 (52%)	394 (98%)	8 (2%)		Reference category
Agree	142 (18%)	130 (92%)	12 (8%)		**0.2**	**(*****p*** **=** **0.001)**
Indifferent	112 (14%)	76 (68%)	36 (32%)		**0.04**	**(*****p*** **<** **0.001)**
Disagree	32 (4%)	15 (47%)	17 (53%)		**0.02**	**(*****p*** **<** **0.001)**
Absolutely disagree	38 (5%)	20 (53%)	18 (47%)		**0.22**	**(*****p*** **<** **0.001)**
Missing information	50 (6%)	49 (98%)	1 (2%)		0.9	(**p** = 0.996)
Once vaccinated, no need to take precautions (wearing mask, social distancing): *N* (%)				0.155		
Absolutely agree	36 (5%)	33 (92%)	3 (8%)		Reference category
Agree	72 (9%)	67 (93%)	5 (7%)		1.2	(*p* = 0.795)
Indifferent	212 (27%)	189 (89%)	23 (11%)		0.8	(*p* = 0.650)
Disagree	181 (23%)	164 (91%)	17 (9%)		0.9	(*p* = 0.841)
Absolutely disagree	265 (34%)	223 (84%)	42 (16%)		0.5	(*p* = 0.245)
Missing information	10 (1%)	8 (80%)	2 (20%)		0.4	(*p* = 0.309)
COVID-19 vaccine is safe: *N* (%)				**<** **0.001**		
Absolutely agree	228 (29%)	223 (98%)	5 (2%)		Reference category
Agree	178 (23%)	172 (97%)	6 (3%)		0.6	(*p* = 0.472)
Indifferent	251 (32%)	218 (87%)	33 (13%)		**0.2**	**(*****p*** **<** **0.001)**
Disagree	52 (7%)	30 (58%)	22 (42%)		**0.03**	**(*****p*** **<** **0.001)**
Absolutely disagree	38 (5%)	12 (32%)	26 (68%)		**0.01**	**(*****p*** **<** **0.001)**
Missing information	29 (4%)	29 (100%)	0 (0%)		NA	
Still need to be vaccinated even if I had a previous COVID-19 infection: *N* (%)				**<** **0.001**		
Absolutely agree	351 (45%)	342 (97%)	9 (3%)		Reference category
Agree	125 (16%)	120 (96%)	5 (4%)		0.6	(*p* = 0.418)
Indifferent	167 (22%)	135 (81%)	32 (19%)		**0.1**	**(*****p*** **<** **0.001)**
Disagree	38 (5%)	23 (61%)	15 (39%)		**0.04**	**(*****p*** **<** **0.001)**
Absolutely disagree	49 (6%)	18 (37%)	31 (63%)		**0.02**	**(*****p*** **<** **0.001)**
Missing information	46 (6%)	46 (100%)	0 (0%)		NA	
COVID-19 vaccines do protect against latest COVID-19 variants: *N* (%)				**<** **0.001**		
Absolutely agree	117 (15%)	114 (97%)	3 (3%)		Reference category
Agree	182 (24%)	178 (98%)	4 (2%)		1.2	(*p* = 0.838)
Indifferent	338 (44%)	292 (86%)	46 (14%)		**0.2**	**(*****p*** **=** **0.003)**
Disagree	63 (8%)	50 (79%)	13 (21%)		**0.1**	**(*****p*** **=** **0.001)**
Absolutely disagree	65 (8%)	39 (60%)	26 (40%)		**0.04**	**(*****p*** **<** **0.001)**
Missing information	11 (1%)	11 (100%)	0 (0%)		NA	

a *Five persons (out of the total of 781 who responded to the questionnaire) did not indicate if they had been vaccinated or not and were excluded from analysis*.

b *p < 0.05 indicates a significant difference (highlighted in bold)*.

### Reasons Given by Hospital Employees Who Chose to Be Vaccinated

The reason employees most frequently chose to be vaccinated included the desire to have their life “back to normal” (*n* = 626, 92%), and being convinced by scientific results discussed in the media (*n* = 471, 69%). Other reasons included knowing someone who had suffered from a complicated COVID-19 infection (*n* = 238, 35%), because themselves or someone close were at high risk of COVID-19 complications (*n* = 131, 19%) and finally, because they had been convinced by an information session at the Hospital (*n* = 100, 15%). Most participants (*n* = 554; 81%) responded that they would recommend the COVID-19 vaccine to a colleague (see Table 4).

**Table 4:** Vaccinated collaborators: attitude towards COVID-19 vaccination.

**(A) Reasons given by collaborators who chose to be vaccinated**	**Total**
**(Several options possible)**	**(*N* = 684, 100%)**
Desire to “get back to normal” outside work (travel, see friends)	626 (92%)
Convinced by scientific results shared by media	471 (69%)
Know someone who has suffered from a severe COVID-19 disease	238 (35%)
Household contacts and/or myself at high risk of COVID-19 complications	131 (19%)
Convinced by information session in hospital	100 (15%)
Has been recommended by a colleague	91 (13%)
Has been recommended by hierarchical superiors	87 (13%)
Felt constrained by hierarchical superiors	12 (2%)
**(B) Recommend COVID-19 vaccine**	**Total**
	**(*****N*** **=** **684, 100%)**
Yes	554 (81%)
No	120 (18%)
Missing information	10 (1%)

### Reasons Given by Hospital Employees Who Refused to Be Vaccinated

Among those who refused to be vaccinated (Table 5A), the most common reasons were that they did not feel at risk of complications (38%), or had questions or concerns that they would like to address to a specialist (35%). Further, 26% felt protected because they had already contracted the disease before. Twenty-four percent reported that they had not been convinced by an information session. Other common reasons were concerns about the COVID-19 vaccine side effects shared by media (22%); because had experienced serious side effects following another vaccine in the past (11%), or a lack of recommendations from their personal doctor (8%). Around half of the non-vaccinated participants said that they would not change their mind (52%). The unvaccinated participants were also asked to expand on which elements would make them change their minds in favour of vaccination (Table 5B). The following responses were provided: more reliable information on vaccine efficacy (12 participants); scientific results showing low risk of side effects (9 participants); mandatory vaccination for certain situations (e.g., travel; end of their pregnancy) (3 participants); co-workers/friends or relatives being vaccinated (1 participant) (Table 5C).

**Table 5:** Non-vaccinated collaborators: attitude towards COVID-19 vaccination.

**(A) Reasons for refusing vaccination**	**Total**
**(Several options possible)**	**(*N* = 92, 100%)**
Do not feel at danger of complicated or severe COVID-19	35 (38%)
Still have questions or concerns that would like to address to a specialist	32 (35%)
Feel protected because had COVID-19 disease before	24 (26%)
Have not been convinced by information session at HUG	22 (24%)
Worried or afraid of side effects shared by media	20 (22%)
Have experienced important side effects after another vaccine in the past	10 (11%)
Was not recommended to you by your personal doctor	9 (8%)
Do not know anyone who has suffered from complicated or severe COVID-19	6 (7%)
Was not recommended by a colleague	4 (4%)
Was not strongly recommended by hierarchical superiors	3 (3%)
**(B) Is there anything that could change your mind and make you choose to be vaccinated**	**Total**
	**(*****N*** **=** **92, 100%)**
Yes	9 (10%)
No	48 (52%)
Rather no	31 (34%)
Missing information	4 (4%)
**(C) If no/rather no: reasons that may change participants' mind**	**Total**
**(Open answers)**	**(*****N*** **=** **79, 100%)**
Will not change mind	45 (57%)
More reliable information on vaccine efficacy	12 (15%)
Mandatory vaccination for certain situation (e.g., travel)	11 (14%)
Scientific results showing low risk of long-term side effects	9 (11%)
Change in my health situation (e.g., end of pregnancy)	4 (5%)
Scientific results showing low risk of side effects	3 (4%)
Co-worker, friends, or relatives getting vaccinated	1 (1%)
Missing information	8 (10%)

## Discussion

In the present study, we report high acceptance of the COVID-19 vaccination among employees of our department (88% of respondents to the survey have been vaccinated). In comparison, by June 25, 2021, the date of the end of our survey, 64% (two doses) and 66% (one dose) of the hospital employees had received COVID-19 vaccines, compared to the 36% (two doses), and 15% (one dose) in the general population of Geneva (3).

Among hospital employees, vaccine acceptance was higher among doctors compared to nurses, and also among those having worked for a longer time in the hospital. Previous reports have also shown that the trust in COVID-19 vaccine increases with the level of education, and a better understanding of the vaccine properties (12–15). In particular, communication/video tutorials on vaccine properties and herd immunity may reduce vaccine hesitancy (13, 14). It is also expected that doctors may be better able to differentiate between information and misinformation due to their medical training. Thus, vaccine acceptability has been reported to be higher among physicians in comparison to other categories of HCW (1, 6).

Further, those who suffered from a chronic disease and had a household contact with a chronic disease were more likely to be vaccinated. Similarly, it has been reported earlier that people belonging to the high-risk categories or being in close contact with a high risk person (i.e., seniors, immunocompromised people, people suffering from chronic conditions) were more willing to be vaccinated (2, 16). However, surprisingly only 19% of the vaccinated hospital employees reported to have chosen the vaccination because they were at higher risk of severe COVID-19 or because they were close to a high risk persons.

Generally, our study found that those with higher awareness of potential risk associated to COVID-19 and who perceived themselves at risk of COVID-19 infection were more likely to accept the vaccine (17). A similar tendency was noted with the fear of being spreaders of infection for patients or for family. The majority of the vaccinated HCW in our survey responded that they would recommend the vaccine to others, as previously reported (4).

Overall, in our study, the main reported reason for reluctance to be vaccinated was a lack of awareness of potential danger of COVID-19 for themselves, and fears and concerns about the vaccine that they would like to address to a specialist. Other reports have observed that vaccine hesitancy was caused by a lack of comprehensive and trustable data, but also because of media controversy (2, 6, 18, 19). All these observations suggest that sufficient investment in vaccine education appears to be urgently needed (2).

The results of this survey suggest that hospital employees share some of the same concerns about COVID-19 vaccines as the general public, including a general lack of knowledge on ARN messenger vaccines and a fear about their safety profiles. Finally, an important proportion of the unvaccinated participants responded that they still had questions or concerns that they wanted to address to a specialist, and others responded that they were not vaccinated because this had not been recommended by their personal doctor. This underlines the role that doctors play in this pandemic to transmit their knowledge and comprehension of COVID-19 vaccines to non-medical colleagues, and also the role of general practitioner to recommend the vaccine to their patients, as reported previously (6, 11, 20, 21).

Our study has several limitations. With a response rate <50%, the results may not be fully representative of all hospital employees at our department. While there is sample selection with over-representation of vaccinated respondents, the results still provide valuable information on the relative vaccination likelihoods of different sub-groups. Under the assumption that groups with lower vaccination rates are less likely to respond to the survey, actual between-group differences may be even larger than what we detect. Hence, if anything, the current results may correspond to an under-estimation of the actual effects. Another limitation is that our findings may not be easily extended to other hospital settings, as national health policies vary between countries (e.g., the current vaccine promotion policies at the federal level in Switzerland differ from policies put in place in neighbouring France or Germany).

## Conclusion

In the present study we report high acceptance of the COVID-19 vaccination among employees of the DPGO in HUG. Nevertheless, several interventions to increase the understanding of the potential danger of SARS-CoV-2 and of the safety of COVID-19 vaccines can be implemented to increase the acceptance of the vaccine among hospital employees. Better trained and informed HCWs could encourage the general population to be vaccinated.

## Data Availability Statement

The raw data supporting the conclusions of this article will be made available by the authors, without undue reservation, on request.

## Ethics Statement

The studies involving human participants were reviewed and approved by Regional Research Ethics Committee (CCER) of Geneva, Switzerland. Written informed consent for participation was not required for this study in accordance with the national legislation and the institutional requirements.

## Author Contributions

AP, KP-B, NW, and GB-R conceived and designed the questionnaire. AP, DR, and GB-R analysed the data. AP and GB-R wrote and revised the paper. All authors have approved the manuscript and agreed with submission to your journal.

## Conflict of Interest

The authors declare that the research was conducted in the absence of any commercial or financial relationships that could be construed as a potential conflict of interest.

## Publisher's Note

All claims expressed in this article are solely those of the authors and do not necessarily represent those of their affiliated organizations, or those of the publisher, the editors and the reviewers. Any product that may be evaluated in this article, or claim that may be made by its manufacturer, is not guaranteed or endorsed by the publisher.
