# Supplementary material for: Acceptability of COVID-19 Vaccine Among Hospital Employees in the Department of Paediatrics, Gynaecology and Obstetrics in the University Hospitals of Geneva, Switzerland
Source: Front Public Health. 2022 Jan 27;9:781562. doi: 10.3389/fpubh.2021.781562 (PMC8830590; doi:10.3389/fpubh.2021.781562)
Supplement: Supplementary file 1 [file Data_Sheet_1.docx]

Questionnaire about Sars-CoV2 and the COVID-19 vaccine

Please tick each of the boxes that correspond to your answer(s):

**Section 1. Few questions concerning Sars-CoV2:** (1 answer only)

1. What is your perception of Sars-Cov2 disease in adults?

□ Not at all severe □ Not severe □ Mild □ Severe □ Very severe

2. What is your perception of Sars-Cov2 disease in infants and children?

□ Not at all severe □ Not severe □ Mild □ Severe □ Very severe

3. What is your perception of Sars-Cov2 disease in pregnant women?

□ Not at all severe □ Not severe □ Mild □ Severe □ Very severe

4. What is your perception of Sars-Cov2 disease in risk groups (i.e. asthma, hearth and pulmonary disease, diabetes..)?

□ Not at all severe □ Not severe □ Mild □ Severe □ Very severe

5. While working in the hospital, do you think that you have a high risk of being exposed to Sars-Cov2)? (1 answer only)

□ Not at all severe □ Not severe □ Mild □ Severe □ Very severe

6. While infected by Sars-Cov2, do you perceive yourself as a spreader with patients that you treat?

□ Absolutely agree □ Agree □ Indifferent □ Disagree □ Absolutely disagree

7. While infected by Sars-Cov2, do you perceive yourself as a spreader with yours household contacts?

□ Absolutely agree □ Agree □ Indifferent □ Disagree □ Absolutely disagree

**Section 2. Few questions concerning COVID-19 vaccines:** (1 answer only)

8. COVID-19 vaccines are effective in preventing severe forms of Sars-Cov2 diseases?

□ Absolutely agree □ Agree □ Indifferent □ Disagree □ Absolutely disagree

9. By reaching a sufficient coverage among the population, COVID-19 vaccines could be a cornerstone to end the Sars-Cov2 pandemic?

□ Absolutely agree □ Agree □ Indifferent □ Disagree □ Absolutely disagree

10. Are COVID-19 vaccines safe?

□ Absolutely agree □ Agree □ Indifferent □ Disagree □ Absolutely disagree

11. We can stop taking precaution after being vaccinated (i.e. stop wearing a mask, social distancing...)?

□ Absolutely agree □ Agree □ Indifferent □ Disagree □ Absolutely disagree

12. Should you still set vaccinated If You already had COVID-19?

□ Absolutely agree □ Agree □ Indifferent □ Disagree □ Absolutely disagree

13. Do COVID-19 vaccines protect against the Sars-CoV2 variants?

□ Absolutely agree □ Agree □ Indifferent □ Disagree □ Absolutely disagree

**Section 3. The vaccination status:**

14. Have you chosen to be vaccinated against COVID-19?

No □ Yes □

→ if no, please go directly to question 24.

→ if yes, please continue till question 23 and then go directly to question 37.

**Section 3a. Vaccinated collaborators**

15. Have you chosen to be vaccinated because someone close to you and/or yourself are at high risk of COVID-19 complications (someone with chronic disease or have risk factors)?

No □ Yes □

16. Have you chosen to be vaccinated because you have followed the scientific results discussed in the media that have convinced you that COVID-19 infections can be severe and vaccines are safe?

No □ Yes □

17. Have you chosen to be vaccinated because you were convinced by an information session at the HUG?

No □ Yes □

18. Have you chosen to be vaccinated because you know someone who has suffered from a complicated COVID-19 infections?

No □ Yes □

19. Have you chosen to be vaccinated because you were recommended to it by a colleague?

No □ Yes □

20. Have you chosen to be vaccinated because you were recommended to it by your hierarchical superiors?

No □ Yes □

21. Have you chosen to be vaccinated because you felt constrained by your superiors?

No □ Yes □

22. Have you chosen to be vaccinated because you want your life “get back normal” outside of work (i.e. travel, see friends…)?

No □ Yes □

23. Do You recommend the COVID-19 vaccine?

No □ Yes □

**Section 3b. Non-vaccinated collaborators**

24. Do you plan to get vaccinated in the next month?

No □ Yes □

25. Have you chosen not to get vaccinated because you don't know anyone who has suffered from complicated or severe COVID-19 disease?

No □ Yes □

26. Have you chosen not to get vaccinated, because you don’t feel in danger of having a complicated or severe COVID-19 disease?

No □ Yes □

27. Have you chosen not to get vaccinated, because you think you are protected as having already contracted the disease?

No □ Yes □

28. Have you chosen not to get vaccinated, because you were not convinced by an information session at the HUG?

No □ Yes □

29. Have you chosen not to get vaccinated, because you were not recommended to it by your personal doctor?

No □ Yes □

30. Have you chosen not to get vaccinated, because it was not recommended to you by a colleague?

No □ Yes □

31. Have you chosen not to get vaccinated, because it was not strongly recommended to you by your superiors?

No □ Yes □

32. Have you chosen not to get vaccinated, because you have experienced serious side effects following a vaccine in the past?

No □ Yes □

33. Have you chosen not to get vaccinated, because you have experienced serious side effects following the first dose of COVID-19 vaccine?

No □ Yes □

34. Have you chosen not to get vaccinated, because you still have questions or concerns that you would like to address to a specialist?

No □ Yes □

35. Do you currently advise your colleagues not to be vaccinated against COVID-19?

No □ Yes □

→ if no, why not? (Open answer)

36. Is there anything that could change your mind and let you get the COVID-19 vaccine?

No □ Yes □

→ if yes, reasons to change in intention to get vaccinated (Open answer)

**Section 4. Demographic questions**

37. What is your gender?

Male □ Female □

38. How many years have you worked in a hospital? (1 answer only)

□ < 1 year □ 1-5 years □ 6-10 years □ > 10 years

39. What is your working category in HUG? (1 answer only)

□ Nurse □ Doctor □ Auxiliary nursing staff □ Technician □ Administrator

□ Therapist □ Social category □ Other category (define which one)

41. You work in contact with (Please tick all answers that apply):

□ New born □ Toddler □ Immunocompromised □ Pregnant □ No one

42. Do you and/or your household contacts suffer from a chronic disease?

No □ Yes □

43. Do you live with children? (Please tick all answers that apply)

□ No □ Yes, < 12 years □ Yes, 12-18 years □ Yes, >18 years
